# Sense of coherence and strategies for coping with stress among nurses

**DOI:** 10.1186/s12912-021-00631-1

**Published:** 2021-06-23

**Authors:** Katarzyna Betke, Małgorzata Anna Basińska, Anna Andruszkiewicz

**Affiliations:** 1 Nicolaus Copernicus University in Poland Ludwig Rydygier Collegium Medicum in Bydgoszcz, Faculty of Health Sciences, Bydgoszcz, Poland; 2 Kazimierz Wielki University in Bydgoszcz, Faculty of Psychology, Bydgoszcz, Poland

**Keywords:** Stress, Nurses, Coping strategies, Work environment, Stress coping strategies, The sense of coherence

## Abstract

**Background:**

The nursing profession is associated with constant presence of difficult situations and stress, which arise from responsibility for the highest values – human life and health. With demographic changes in the society, the demand for nursing care increases. Looking after the health of nursing staff becomes a non-material investment in employees. One of the most important health potentials described in literature is the sense of coherence. It can significantly contribute to maintaining good health, modify one’s functioning in a stressful working environment and influence the choice of strategies for coping with stress.

**Aim:**

The aim of the study was to describe the specific relationship between the sense of coherence and strategies for coping with stress in a group of professionally active nurses.

**Methodology and methods:**

The study involved 91 nurses in central Poland, aged 22–52. The group was diversified in terms of: education, work system, marital status and place of residence. The study was conducted with the use of: Sense of Coherence Questionnaire SOC-29 and Inventory to Measure Coping Strategies with Stress - Mini-COPE. The study was conducted in accordance with the principles of scientific research set out in the Helsinki Declaration.

**Results:**

The sense of coherence value in the test group was M = 134.24 (SD = 19.55). In stressful situations nurses most often used active strategies to cope with stress: Planning M = 2.10 (SD = 0.54), Seeking Emotional Social Support M = 1.95 (SD = 0.68) and Seeking Instrumental Social Support M = 1.95 (SD = 0.69), and the least frequently: Alcohol/Drug Use M = 0.28 (SD = 0.48). The level of the sense of coherence and its components differentiated the strategies of coping with stress used in the examined group of nurses.

**Conclusions:**

The research confirmed that the sense of coherence serves as a health potential in a stressful working environment - a high sense of coherence translate into better mental health, correct functioning in the working environment, and using adaptive strategies of coping with stress. Nurses with a stronger sense of coherence used more adaptive strategies to deal with stress than those with average or low levels.

**Supplementary Information:**

The online version contains supplementary material available at 10.1186/s12912-021-00631-1.

## Background

Nursing is a profession commonly considered difficult and not always appreciated by the society, but there is a great need for it [1–3]. Twenty-first century nurses face challenges such as different job opportunities, nurse shortages, negative effectiveness, workload concerns, system change, and increasing complexity of clinical practices. In these situations, they must be able to respond in a timely, effective, creative and flexible manner [4]. Nurses are the largest medical professional group. They are individuals with higher education degrees, who make their own decisions concerning the care administered to patients and who provide a wide range of health services. They carry out their everyday tasks in continuous contact with other people, which is linked to holding responsibility for the highest values: human life and health. At the same time, apart from the duties performed, more expectations are set by their superiors, other members of the therapeutic team, as well as patients and their relatives.

The European Agency for Safety and Health at Work and the results of worldwide studies place nursing among the most stressful professions [5]. Numerous studies have shown that nurses very often consider their work stressful [6–9]. The sources of stress in the work of a Polish nurse include:

- *stress related to the workplace and its conditions* (organizational chaos and overload with professional duties, excess work, shifts, low pay - disproportionate to the effort, exposure to factors potentially harmful to physical and mental health),
- *stress related to relationships with colleagues* (atmosphere in the therapeutic team, interpersonal relationships, conflicts among nurses and other members of the therapeutic team),
- *stress related to the contact with the patient and their family* (too many patients, problems of the patient and their family resulting from the disease, exposure to serious infectious diseases (e.g. SARS-CoV-2 infection, HIV), professional problems resulting from working with aggressive, agitated, and dying patients [6].

The sources of stress are similar to the burden in the workplace of nurses around the world [7, 8]. The profession is also associated with exposure to harmful biological, chemical, physical and psychological factors, as well as stressful cooperation with patients and their families [9–15]. Long-term functioning in a stressful working environment has an undeniable impact on health. It also triggers the need for specific actions, which are defined as coping strategies. Coping with stress, according to the authors of the transactional stress theory - Richard Lazarus and Susan Folkman - is a constantly changing cognitive and behavioural effort, focused on specific external and internal struggles, in situations seen as burdensome or exceeding personal capabilities. In order to cope with difficulties, a person can choose active ways of solving problems, considered pro-health or evasive strategies. Dealing with stress can be considered a behaviour significant for human well-being. The use of personal resources has an effect on health, well-being and effective functioning in the work environment [16]. The choice of adaptive strategies for coping with stress can also be influenced by a generalised and relatively stable dispositional orientation, which is characteristic for a given individual and which determines the fundamental way of perceiving, feeling and understanding the world around and one’s own life, i.e. the so-called Aaron Antonovsky’s sense of coherence (SOC). There are three correlated components of the sense of coherence: the sense of comprehensibility, the sense of manageability and the sense of meaningfulness [17]. According to Aaron Antonovsky, “the key to health”, i.e. the main factor thanks to which a person can cope with stress and not fall ill or can recover faster in case of illness is precisely the sense of coherence [17–20]. The functional aspect of the sense of coherence is most noticeable in situations of stress and its management. The role of this construct is to prevent transformation of a state of tension into a state of stress, as well as to promote the use of more adaptive strategies for coping with difficult situations [21]. The sense of coherence influences the way of perceiving stressful situations occurring during the performance of professional tasks, favours engaging in problem-oriented strategies and also plays a role in protecting employees from the negative effects of stress experienced. Studies conducted in a group of over a thousand nurses in Poland have shown that a strong sense of coherence reduces the level of experienced stress, irrespective of the strength of the stressors and weakens its negative effects [18, 22]. The current situation, i.e. a shortage of nursing staff, the ageing of this professional group and the fact that nursing graduates do not take up this kind of employment creates a need to look for solutions to these problems. According to Małgorzata Anna Basińska and Anna Andruszkiewicz, it seems justified to take actions aimed at reducing stressors in everyday work, as well as to develop and strengthen personal resources and the ability to cope with difficult situations. Creating a friendly work environment, strengthening health meta-resources, as well as providing training aimed at developing an ability to choose constructive strategies for coping with stress should become a permanent component of investment in employees [23]. It seems necessary to implement a strategy to prevent stress and its consequences in the workplace. Today, stress should not be seen as a weakness, but rather as a phenomenon that can be managed by creating a culture of openness and understanding [24].

The aim of the study was to describe the specific relationship between the sense of coherence and strategies for coping with stress in a group of professionally active nurses.

## Methods

### Study design and participants

This cross-sectional study was to describe the specific relationship between the sense of coherence and strategies for coping with stress in a group of professionally active nurses. 91 nurses in central Poland participated in this study.

### Procedure

The data was collected from November 2015 until June 2016. The questionnaire packages were made available to nurses during postgraduate studies. The persons interested in the study were informed that in the designated place there are sets of questionnaires intended for the study. People willing to take part in the study, after completing the sets, could place them in a specially secured urn. The return of the questionnaires was tantamount to giving informed consent to participate in the study. A total of 91 respondents took part.

#### Measurment

##### Sense of coherence

The Sense of Coherence Questionnaire (SOC-29) by Antonovsky, published in 1983, was used to measure the sense of coherence. To measure the sense of coherence, the Sense of Coherence Questionnaire (SOC-29) by Antonovsky was used, in an adapted Polish version (Koniarek et al., 1993). The questionnaire allows to estimate the level of the sense of coherence, as well as the levels of its three components: the sense of comprehensibility (SOC comprehensibility), manageability (SOC manageability) and meaningfulness (SOC meaningfulness).

The SOC-29 questionnaire consists of 29 test items, expressed in the form of interrogative sentences. Each item is graded on a 7-point rating scale. The overall score is obtained by summing all the values. The questionnaire consists of three subscales corresponding to the components of the sense of coherence: comprehensibility, containing 11 questions, manageability - controllability, consisting of 10 questions, and meaningfulness including 8 questions. In order to assess the sense of comprehensibility, the respondent answers the question: “When you talk to people, do you have the feeling that they don’t understand you?” (1 – never, 7 – always have this feeling). Example question about the sense of manageability is “When you think about your life, you very often” (1 - feel how good t is to be alive, 7 - ask yourself why you exist at all). The sense of meaningfulness evaluates the question: “How often do you have the feeling that there’s little meaning in the things you do in your daily life?” (1 - very often, 7 - very seldom/never). The results are calculated using appropriate keys that allow to define the sense of coherence and its components (Antonovsky, 1993). The Sense of Coherence Questionnaire is listed as Supplementary File 1.

The evaluation of the Polish version of the SOC-29 Questionnaire showed a very high reliability of the tool. The internal consistency indices, calculated using the half-method with the Sperman-Brown correction, amounted to 0.92 for the sense of coherence, 0.78 for the sense of comprehensibility 0.78, for the sense of manageability 0.72 and for the sense of meaningfulness 0.68, respectively, while the internal consistency expressed in the Cronbach’s alpha index was 0,78. The stability over time of the scale ranged from 0.72 to 0.83 (*p* < 0.001) [17, 22].

#### Strategies for coping with stress

To evaluate strategies for coping with stress, the Inventory to Measure Coping Strategies with Stress (Mini-COPE) by Carver, Scheier and Weintraub was used, in an adapted Polish version (Juczyński, Ogińska-Bulik, 2009).

The inventory is a tool for assessing the stress coping strategies used. It is an abridged version of the Multidimensional Coping Inventory-COPE. The questionnaire consists of 28 statements included in 14 strategies of coping with stress, including: Active Coping, Planning, Positive Reinterpretation and Growth, Acceptance, Humour, Religion, Seeking Emotional Social Support, Seeking Instrumentalal Social Support, Suppression of Competing Activities, Denial, Restraint Coping, Alcohol/Drug Use, Behavioural Disengagement. Each scale consists of 2 items. The respondent responds to each statement on a scale from 0 (I almost never do it) to 3 (I almost always do it). Sample questions: “‘I’ve been concentrating my efforts on doing something about the situation I’m in” or “I’ve been using alcohol or other drugs to help me get through it” (1 - I haven’t been doing this at all, 4 - I’ve been doing this a lot). The full version of Inventory to Measure Coping Strategies with Stress (Mini-COPE) is presented in Supplementary File 2.

The Polish version is characterized by high reliability, internal consistency expressed in the Cronbach’s alpha index was 0.78 for the entire scale and from 0.92 to 0.66 for the subscales [22].

#### Participants characteristic

The study involved 90 female nurses and 1 male nurse aged 22–52 (M = 38, SD = 7.46), the group was diversified in terms of socio-demographic variables and variables connected to professional work. The majority of respondents declared their marital status to be “in a relationship”, the least of them were “separated” or “widowed”. The majority of the respondents had higher education vocational degrees, the average number of years in the profession was over 16 years (M = 16.597; SD = 8.35), and the majority of the respondents were unit nurses. A self-authored questionnaire was used to collect socio-demographic data from the study group, which was presented as Supplementary File 3. The study was conducted in accordance with the principles of scientific research set out in the Helsinki Declaration. The research was approved by the Bioethics Committee of October 20, 2015 (KB 666/2015). The individuals participating in the study on a voluntary basis were healthy and did not have a professional or other relationship with the researcher.

## Results

### Data analysis

For the analysis of the obtained research results, statistical methods were used, which made it possible to answer the formulated research goal. The analysis of the obtained results was performed using the Statistica 9.0 program. In return 91 complete questionnaires were received.

The analyses presented below were performed on the basis of a correlation model. The following statistical tests were used:

1. To describe group variables the mean (M) and standard deviation (SD), and number (f) and percentage (%)
2. To determine the direction and strength of correlation between variables Pearson’s linear correlation test (when there was a linear correlation between variables) or Spearman’s nonparametric test (when variables were qualitative)
3. To assess the significance of differences between multiple means ANOVA variance analysis or ANOVA rank analysis by Kruskal-Wallis (in the absence of a normal distribution)
4. NIR test; to examine the significance of differences between individual variables

The first step of the analysis was to calculate average values for all variables. The results obtained were presented in the form of raw average results. Individual tables differ in terms of the number of presented studies due to exclusion of incomplete questionnaires from the analyses. The studied group of nurses obtained an average value of the sense of coherence of M = 134.24. The values of the sense of coherence and its components were highly differentiated, which is presented in Table 1.

**Table 1:** Mean raw scores (M), standard deviation (SD), in terms of sense of coherence and its components (*N* = 88) in the studied group of nurses

Dimensions sense of coherence	M	SD	Minimum	Maximum
**Sense of coherence**	134.24	19.551	79.00	196.00
**Sense of comprehensibility**	44.56	9.190	23.00	77.00
**Sense of mangeability**	47.24	8.203	24.00	63.00
**Sense of meaningfulness**	42.44	5.952	26.00	56.00

The nurses from the study group most often declared the use of strategies classified as active ways of coping with stress, focused on the problem. The results are presented in Table 2.

**Table 2:** Average raw results (M), standard deviation (SD) in the scope of coping with stress strategies (*N* = 91) in the studied group of nurses

Strategies for coping with stress	M	SD	Minimum	Maximum
**Active Coping**	**2.19**	**0.475**	**1.00**	**3.00**
**Planning**	**2.10**	**0.540**	**0.50**	**3.00**
**Positive Reinterpretation and Growth**	**1.82**	**0.593**	**0.00**	**3.00**
**Acceptance**	1.71	0.558	0.50	3.00
**Humour**	0.65	0,575	0.00	3.00
**Religion**	1.30	0.937	0.00	3.00
**Seeking Emotional Social Support**	**1.95**	**0.681**	**0.00**	**3.00**
**Seeking Instrumental Social Support**	**1.95**	**0.693**	**0.00**	**3.00**
**Suppression of Competing Activities**	1.46	0.720	0.00	3.00
**Denial**	0.91	0.649	0.00	3.00
**Restraint Coping**	1.33	0.620	0.00	2.50
**Alcohol/Drug Use**	0.28	0.484	0.00	2.00
**Behavioural Disengagement**	0.68	0.569	0.00	2.00

The next stage of the analysis was to answers the question of: Does the level of the sense of coherence and its components differentiate the strategies of coping with stress used in the examined group? The nurses were divided into three subgroups based on the results obtained: individuals with a low sense of coherence, with an average sense of coherence and with a high sense of coherence according to the criterion (mean +/− ½ SD).

### The sense of coherence and the strategies used to cope with stress

The results of the analyses show that the sense of coherence significantly differentiates the studied group in terms of the applied coping strategies, they were presented in Table 3.

**Table 3:** Sense of coherence and strategies for coping with stress (*N* = 88) – results of analysis of variance

Strategies for coping with stress	Statistical test F / H	P
**Active Coping**	0.44	0.645
**Planning**	0.19	0.826
**Positive Reinterpretation and Growth**	2.59	0.081
**Acceptance**	0.99	0.375
**Humour**	1.95	0.148
**Religion**	1.72	0.185
**Seeking Emotional Social Support**	3.06	0.052
**Seeking Instrumental Social Support**	0.90	0.411
**Suppression of Competing Activities**	0.40	0.670
**Denial**	**7.58**	**0.001**
**Restraint Coping**	1.58	0.211
**Alcohol/Drug Use**	H (2, *N* = 88) = 5.150	0.076
**Behavioural Disengagement**	**5.32**	**0,007**
**Focus on and Venting of Emotions**	**7.46**	**0.001**

Nurses belonging to the subgroup with a high sense of coherence used Denial, Behavioural Disengagement and Focus on and Venting of Emotions strategies less frequently than the subgroup with low and average sense of coherence. The respondents from the subgroup with a high sense of coherence were significantly less likely to use the Alcohol/Drug Use strategy than those from the subgroup with a low sense of coherence, who most often declared their choice of this strategy. Differences in the application of the Alcohol/Drug Use strategy between individuals from the particular subgroups in terms of the sense of coherence is presented in Table 4.

**Table 4:** Differences in the application of strategies for coping with stress between individuals from the particular subgroups in terms of the sense of coherence and its components

Strategies for coping with stress	Level sense of coherence		
	*Low sense of coherence*	*Average sense of coherence*	*High sense of coherence*
Alcohol/Drug Use	M (0,42)	M (0,34)	M (0,16)
	*Low sense of comprehensibility*	*Average sense of comprehensibility*	*High sense of comprehensibility*
Religion	M (1,38)	M (1,36)	M (0,78)
Focus on and Venting of Emotions	M (1,58)	M (1,58)	M (0,98)
	*Low sense of meaningfulness*	*Average sense of meaningfulness*	*High sense of meaningfulness*
Seeking Instrumental Social Support	M (0,62)	M (2,18)	M (1,94)
Alcohol/Drug Use	M (0,52)	M (0,28)	M (0,18)

### The sense of comprehensibility and the strategies used to cope with stress

The result in Table 5 show that the sense of comprehensibility significantly differentiates the studied group in terms of the applied coping strategies.

**Table 5:** Sense of comprehensibility and strategies for coping with stress- analysis of variance

Strategies for coping with stress	Statistical test F / H	P
**Active Coping**	0.193	0.825
**Planning**	0.027	0.974
**Positive Reinterpretation and Growth**	2.518	0.087
**Acceptance**	0.507	0.604
**Humour**	2.997	0.055
**Religion**	**H = 11.800**	**0.003**
**Seeking Emotional Social Support**	0.910	0.407
**Seeking Instrumental Social Support**	1.133	0.327
**Suppression of Competing Activities**	0.382	0.684
**Denial**	**6.822**	**0.002**
**Restraint Coping**	3.047	0.053
**Alcohol/Drug Use**	2.746	0.070
**Behavioural Disengagement**	1.490	0.231
**Focus on and Venting of Emotions**	**H = 13.635**	**0.001**

Nurses from the subgroup with high levels of comprehensibility were significantly less likely to use the Denial coping strategy than those belonging to the subgroups with low and average levels of comprehensibility.

Nurses from the subgroup with high levels of comprehensibility were significantly statistically less likely to use the Religion and Focus on and Venting of Emotions coping strategy than those belonging to the subgroups with low and average levels of comprehensibility. The results are presented in the Table 4.

### The sense of manageability and the strategies used to cope with stress

The results in Table 6 show that the sense of manageability significantly differentiates the studied group in terms of the applied coping strategies.

**Table 6:** The sense of mangeability and used to strategies for coping with stress

Strategies for coping with stress	Statistical test F / H	P
**Active Coping**	H (2, *N* = 88) = .585	0.746
**Planning**	0.49	0.617
**Positive Reinterpretation and Growth**	0.17	0.843
**Acceptance**	0.46	0.635
**Humour**	0.18	0.833
**Religion**	H (2, *N* = 88) = 3.422	0.181
**Seeking Emotional Social Support**	**H (2,** ***N*** **= 88) = 6.902**	**0.032**
**Seeking Instrumental Social Support**	2.48	0.090
**Suppression of Competing Activities**	1.26	0.289
**Denial**	**3.71**	**0.029**
**Restraint Coping**	0.82	0.444
**Alcohol/Drug Use**	H (2, *N* = 88) = 5.653	0.059
**Behavioural Disengagement**	**3.13**	**0.049**
**Focus on and Venting of Emotions**	**8.66**	**0.000**

Nurses from the subgroup with high levels of manageability were significantly more likely to use the Seeking Emotional Social Support coping strategy than those belonging to the subgroups with low and average levels of manageability. Nurses from the subgroup with a low level of sense of manageability were the least likely to choose this strategy.

Nurses from the subgroup with high levels of manageability were significantly less likely to use the Denial and Behavioural Disengagement coping strategy than those belonging to the subgroup with a low level of manageability. Respondents from the subgroup with high levels of manageability were significantly less likely to choose the Focus on and Venting of Emotions coping strategy than those belonging to the subgroups with low and average levels of manageability.

### The sense of meaningfulness and the strategies used to cope with stress

The results of the analyses in Table 7 show that the sense of meaningfulness significantly differentiates the studied group in terms of the applied coping strategies.

**Table 7:** Sense of meaningfulness and used strategies for coping with stress- analysis of variance

Strategies for coping with stress	Statistical test F / H	P
**Active Coping**	0.030	0.971
**Planning**	0.090	0.914
**Positive Reinterpretation and Growth**	H (2, *N* = 88) = 2.034	0.362
**Acceptance**	0.595	0.554
**Humour**	H (2, *N* = 88) = 4.019	0.134
**Religion**	0,007	0.993
**Seeking Emotional Social Support**	**3.838**	**0.025**
**Seeking Instrumental Social Support**	**H (2,** ***N*** **= 88) = 7.787**	**0.020**
**Suppression of Competing Activities**	1.606	0.207
**Denial**	1.122	0.331
**Restraint Coping**	1.032	0.361
**Alcohol/Drug Use**	**H (2,** ***N*** **= 88) = 3.005**	0.223
**Behavioural Disengagement**	2.432	0.094
**Focus on and Venting of Emotions**	**3.806**	**0.026**

Nurses from the subgroup with high levels of sense of meaningfulness were significantly statistically more likely to use the Seeking Emotional Social Support coping strategy than those belonging to the subgroups with low and average levels of meaningfulness. The respondents from the subgroup with a high level of sense of meaningfulness were less likely to use the Focus on and Venting of Emotions strategy than those with a low and average sense of meaningfulness.

The analysis showed that nurses from the subgroup with an average level of sense of meaningfulness significantly more often chose the strategy of Seeking Instrumental Social Support in difficult situations. Nurses from the subgroup with a low level of sense of meaningfulness were the least likely to choose this strategy. Respondents from the subgroup with a low sense of meaningfulness were significantly more likely to use the Alcohol/Drug Use strategy than those belonging to the subgroups with a high and average sense of coherence. The results are presented in Table 4.

In our study, nurses obtained an average score of the sense of coherence equal to M = 134.24, similar to the results obtained so far in this professional group, both in Poland and in the world. The respondents most often declared the use of strategies classified as active ways of coping with stress, focused on the problem. They most often used Active Coping and Planning. The respondents were the least likely to choose strategies focused on emotions. The analyses confirmed that the sense of coherence significantly differentiated the studied group in terms of the strategies of coping with stress - nurses belonging to the subgroup of people with a high level of sense of coherence and its components used avoidance strategies significantly less frequently. A dangerous tendency to choose the Alcohol / Drug Use strategy was noticed among nurses with the lowest level of sense of coherence.

## Discussion

This study confirmed a correlation between the sense of coherence and coping strategies in the examined group of nurses. These results correspond to conclusions presented by both Polish and foreign researchers [11, 20, 21, 25] concerning the pro-health and buffer role played by the sense of coherence in a stressful working environment.

The studied group presented a varied level of sense of coherence and its components, similarly to the results published in literature. The research on the sense of coherence showed that nurses had a sense of coherence at a level similar to that indicated by Polish researchers - the lowest level of the sense of coherence among nurses was reported by Koniarek (1992): M = 124.60 [26], while the highest - by Kretowicz (2011): M = 145.02 [26]. Polish nurses presented a similar level of the sense of coherence to paediatric nurses in Hong Kong: M = 135.75 [27], in South Africa: M = 137.92 (SD = 20.46) [28], in Brazil: M = 144.60 (SD = 22.60) [29], as well as in Japan: M = 124.40 (SD = 21.20) [30].

The sense of coherence can protect an individual from the destructive effects of difficult and stressful situations. Many studies have shown that a high sense of coherence significantly reduces the effects of stress and helps cope with the difficulties and challenges of life [11, 19, 21, 23, 26, 28, 31]. The components of the sense of coherence also have a significant preventive effect [17, 21]. The sense of coherence determines the way of perceiving stressful situations at work, favours engaging in problem-oriented strategies and also plays a role in protecting employees from the negative effects of stress experienced. Studies conducted in a group of over a thousand nurses in Poland have shown that a high sense of coherence reduces the level of experienced stress, irrespective of the strength of the stressors and weakens the negative effects of stress. The buffering role of the sense of coherence was confirmed primarily in relation with the sense of manageability and the sense of meaningfulness [18, 22].

This is important for nursing staff, whose work is performed in situations of emotional stress and tension. Antonovsky proved that in stressful situations a high sense of coherence encourages more adaptive strategies for coping with difficult situations, i.e. focusing on the task instead of on emotions, and seeking social support [21]. Yam et al. analysed SOC and perceived stress with a sample of critical care nurses, finding that SOC was a protective factor in relation to stress perceptions arising from the work environment. Höge et al. investigated the possible impact of SOC and negative affectivity on the relationship between work stressors and strain. They found a strong correlation between SOC and negative affectivity [32]. Medical Universities in Poland include Antonovsky’s theory into practice, offering, in accordance with current teaching curricula and standards, study modules which help students strengthen their sense of coherence. These include, among others, psychology with elements of interpersonal communication, health promotion, basics of psychotherapy. The process of strengthening the sense of coherence should be continued in the working environment of nursing staff. According to Korcz and Korcz-Biernat, it seems reasonable to take measures that would allow nursing staff to increase their participation in decision-making, also through partnership-based relations with other members of the interdisciplinary team. The authors also emphasize the importance of developing an ability to cope with stress at work [33]. The objectives of salutogenesis are addressed at people who in their everyday work help others and perform duties in conditions of high emotional tension and stress. The author of the theory of salutogenesis stated that his book “Unraveling the Mystery of Health. How People Manage Stress and Stay Well” was also written with nurses in mind, hoping for his theory to be empirically verified by that professional group [17].

The examined group of nurses more often reported choosing pro-health, problem-oriented stress management strategies (i.e. Active Coping, Planning, Seeking Instrumental Social Support and Seeking Emotional Social Support), which corresponds to the results of other authors’ research in this area. Polish nurses most often reported choosing active and constructive actions as a strategy for coping with occupational stress [23]. According to Perek et al., this is a typical way of coping with stress in this occupational group [9]. Active stress management strategies are more commonly used by people with a higher level of coherence. This was confirmed by our own research. Basińska and Andruszkiewicz, using the AVEM Questionnaire and the Latack Scale, studied stress management strategies and work-related experiences among 150 nurses working at different wards. The authors proved that there were no differences between coping strategies chosen by nurses depending on the department they worked at. Those who coped with problems at work through the use of Suppression of Competing Activities/ Behavioural Disengagement strategy used offensive problem-solving strategies less often. Nurses who coped with problems through the Positive Reinterpretation and Growth strategy used more offensive ways of solving problems and were more satisfied with their lives. More often, offensive methods and a task-based mode were used by nurses using Active Coping, Seeking Emotional Social Support and Seeking Instrumental Social Support strategies [23]. The results of the Polish study correspond to the reports of other researchers. Australian nurses, as Lim indicates, choose problem-oriented strategies more often than emotion-oriented ones [34]. Studies on the use of stress management strategies by French nurses indicate that they most often choose Active Coping, Restraint Coping and Positive Reinterpretation and Growth strategies [35]. The study also found that nurses turned to strategies to reduce emotional tension, such as: Seeking Emotional Social Support, Positive Reinterpretation and Growth and Acceptance. The least respondents chose evasive strategies, which are primarily aimed at forgetting or distracting attention from the problem.

The main objective of the study was to assess the correlation between the sense of coherence and the strategies used to deal with stress. These study confirmed the existence of such a correlation. The examined nurses with a high level of coherence were less likely to use evasive strategies, i.e. Denial, Behavioural Disengagement and Focus on and Venting of Emotions, than those with a low and average level of sense of coherence, which has a positive impact on their health and professional functioning. According to Antonovsky, *“people with a strong sense of coherence choose a specific strategy of coping, which in their opinion is most adequate for a given stressor”,* therefore, they use more adaptive techniques to deal with problems that may contribute to better functioning and maintaining good health. They are also able to make optimal use of the resources available to them, updating and flexibly adapting to changing circumstances those coping strategies that prove to be the most effective in a particular situation [36]. Strong SOC is believed to be related to overall human well-being. This relationship was confirmed by Miyata et al., Who showed in nurses a positive relationship between SOC and good mental health and good physical health.

Nurses working in hospitals more often declared the burden of general health problems, as well as symptoms of stress, depression and eating disorders in relation to the general population. SOC turned out to be the most important predictor of general mental health problems and symptoms of post-traumatic stress disorder. It is believed that SOC can play a key role in the onset of these health problems, shaping the perception of and attitudes towards negative work experiences and stress in the work environment [32].. SOC was found to be the most important predictor for general mental health problems and post-traumatic stress symptoms [37]. The choice of stress management strategies that are beneficial to human health has an effect on maintaining health potential and functioning in a stressful work environment. Active strategies make it easier to stay healthy. Evasive strategies may reduce the positive impact of the sense of coherence and make it difficult to maintain good health.

In our study, we also observed an increased tendency for choosing an evasive stress management strategy - Alcohol/Drug Use - by nurses with low levels of coherence. This trend is in line with Antonovsky’s theory. People with a weak sense of coherence more often experience negative emotions such as shame, discouragement, anxiety, which in effect inhibits action-taking. They perceive problems inaccurately, which leads to a failure to make the necessary efforts to cope effectively [18–20, 27].

Empirical research has shown that the sense of coherence and its components correlate with strategies used to deal with stressful situations. A high level of comprehensibility reduces the intensity of assessment of stressful situations, especially those related to threat and loss. The sense of manageability works in a similar way, which additionally influences the perception of the situation as a challenge. Whereas a strong sense of meaningfulness maximizes the perception of stressors as challenges and limits the perception of a situation as a loss. People with a strong sense of meaningfulness take on the challenges of life, search for meaning and make an effort to solve and deal with problems [19, 21].

The study found that nurses with a stronger sense of comprehensibility, manageability and meaningfulness were more likely to choose emotionally focused strategies, and least likely to use non-adaptive strategies, whose consequences may be detrimental to the one’s functioning and health [38]. Research by Isa et al. proved that the nurses surveyed in Brunei often use coping strategies, a pro-health emotional coping strategy. Nurses using strategies of coping with emotions showed higher psychological competences and significantly better professional functioning and personality traits. Emotion-oriented coping strategies are more often used by people who, according to their personality traits, find it easier to undertake and maintain a state of emotional arousal in response to or anticipating emotionally burdensome events. The study confirmed that people who use coping strategies have worse health because they are more likely to use negative styles of coping with emotions, e.g. drinking alcohol and drugs [39]. The sense of meaningfulness, the component Antonovsky treats as a motivational-emotional one, and according to Antonovsky, the most important one in creating the sense of coherence, correlated with the more frequent choice of the Seeking Emotional Social Support strategy, and the least frequently with the choice of the Focus on and Venting of Emotions strategy. What is important, the weaker the sense of meaningfulness of the respondents, the more often they used the Alcohol/Drug Use strategy. The use of this and other evasive strategies by nurses may contribute to deterioration of health, ineffective functioning in the work environment and absenteeism, which in the current situation of shortages in nursing staff is an undesirable situation. In relation to the above, it is justified to implement psycho-educational measures developing an ability to choose adaptive stress management strategies, which should be addressed especially at people with a weak sense of coherence. Strengthening the potential of the sense of coherence is in line with both Antonovsky’s idea and the contemporary trend of positive psychology [40].

Investing in employees means reaching to their internal resources and developing their ability to cope with difficult situations. This investment will contribute to effective operation at work and better quality of services provided to the society. Implementation of strategies aimed at preventing stress and its consequences in the workplace should take place on both a personal and organisational level. Stress management programmes including education and training on stress management are a personal-level strategy that provides support for nurses experiencing stress. An organisation-focused approach addresses work-related stressors by reducing or eliminating them. Long-term implementation of this strategy will allow for better management of nursing staff and for ensuring adequate resources [38].

The presented research results may constitute a source of knowledge for designing psycho-educational programmes addressed both at students - future nurses and active professionals in order to minimize the occurrence of risky behaviours and to help develop skills to cope with them.

### Impact

On international level, the Polish nurses reported similar sense of coherence. Findings from this study will have an impact on how nurses can manage work related stress in terms of sense of coherence. There will also be an impact on nurses’ well-being, which in a long run benefits patients.

### Limitations

The present study has several limitations: Firstly, the relatively small sample of 91 nurses limits the findings’ representativeness. This study limits generalization of findings owing to the small sample size, which might also reduce its power to detect significance. More studies of a prospective nature, with larger sample sizes and that also consider additional variables, such as culture, attitude and beliefs, are required to provide in-depth understanding.

## Conclusions

1. The level of the sense of coherence and its components in the examined group of nurses was similar to results reported for this group to date.

2. The sense of coherence serves as a health potential in a stressful working environment.

3. Nurses with a stronger sense of coherence more often chose adaptive coping strategies, considered pro-health strategies.

4. It is recognised that strengthening health potential i.e. the sense of coherence is justified, as it can significantly modify an individual’s functioning in a stressful working environment.

## Supplementary Information

**Additional file 1.**

**Additional file 2.**

**Additional file 3.**

## Data Availability

The data generated and analysed during the current study are not publicly available due to the sensitivity of the data.
