# Supplementary material for: Sense of coherence and strategies for coping with stress among nurses
Source: BMC Nurs. 2021 Jun 23;20:107. doi: 10.1186/s12912-021-00631-1 (PMC8220127; doi:10.1186/s12912-021-00631-1)

**Sense of Coherence – Orientation to Life Questionnaire**

The Orientation to Life questionnaire has 29 questions regarding different areas in your life. Each question has 7 alternative answers – circle the answer (only one answer) that you feel is the answer closest “to heart” for you. There is no “right” or “wrong” answer so be honest to yourself.


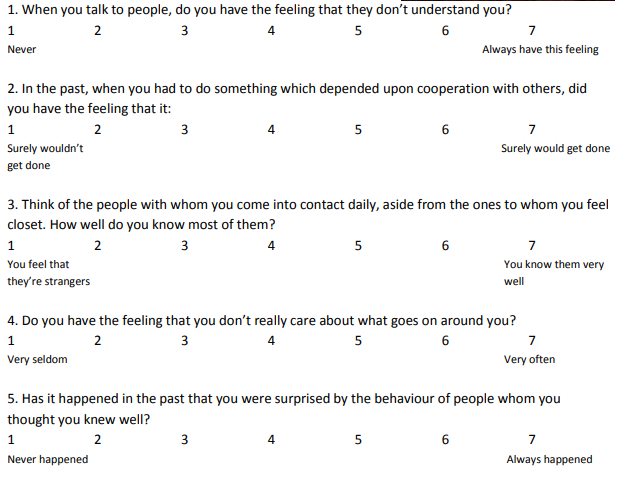


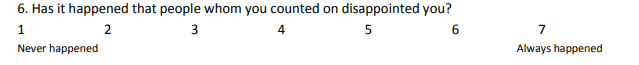


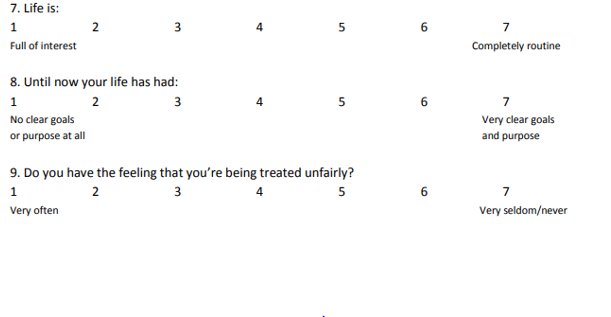


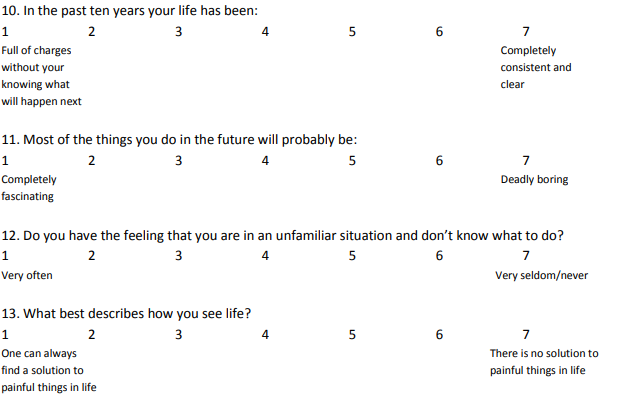


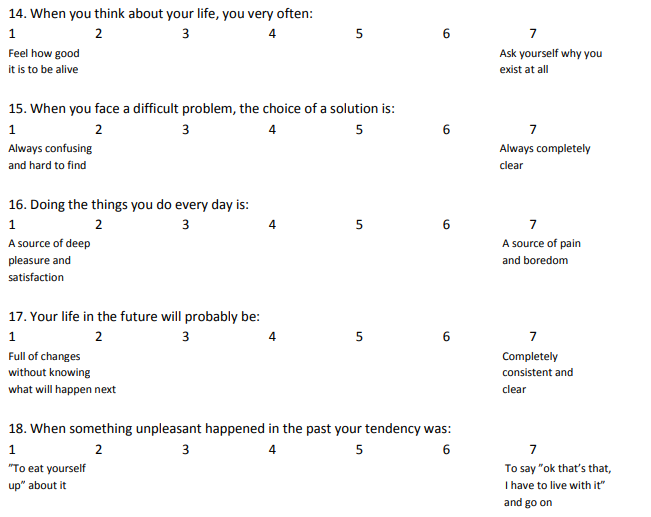


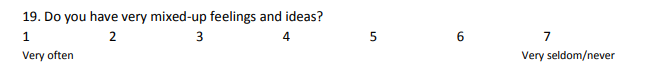


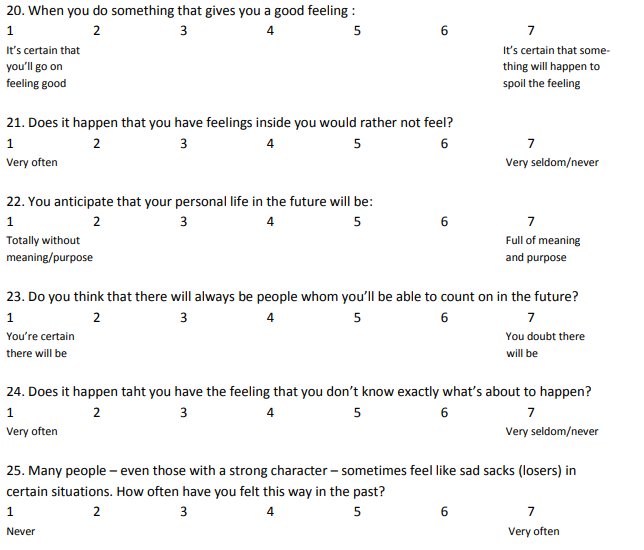


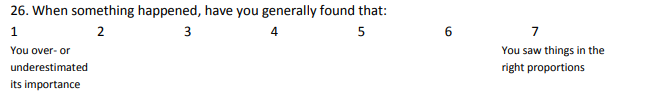


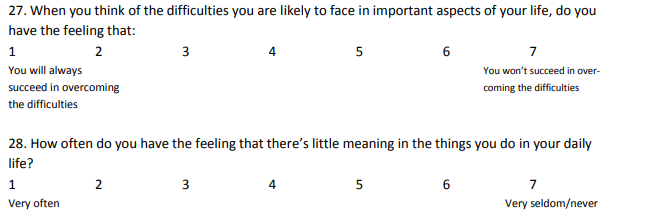


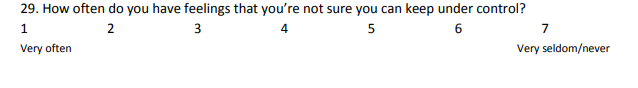

Supplement: Supplementary file 1 — Additional file 1. [file 12912_2021_631_MOESM1_ESM.docx]
