# Supplementary material for: Sense of coherence and strategies for coping with stress among nurses
Source: BMC Nurs. 2021 Jun 23;20:107. doi: 10.1186/s12912-021-00631-1 (PMC8220127; doi:10.1186/s12912-021-00631-1)
Supplement: Supplementary file 2 — Additional file 2. [file 12912_2021_631_MOESM2_ESM.pdf]

Brief COPE (Carver, 1997).

You have done really well – thank you. These next items deal with ways you've been coping with the stress in your life. The stress issue is the ‘it’ in some of the items! There are many ways to try to deal with problems. These items ask what you've been doing to cope with present stresses. Each item says something about a particular way of coping and please avoid answering on the basis of whether how you’ve been coping seems to be working or not—just whether or not you're doing it. Use these response choices and try to rate each item separately in your mind from the others. Make your answers as true FOR YOU as you can.

Coding categories:

1 = I haven't been doing this at all
2 = I've been doing this a little bit
3 = I've been doing this a medium amount
4 = I've been doing this a lot

1. I've been turning to work or other activities to take my mind off things.
2. I've been concentrating my efforts on doing something about the situation I'm in.
3. I've been saying to myself "this isn't real."
4. I've been using alcohol or other drugs to make myself feel better.
5. I've been getting emotional support from others.
6. I've been giving up trying to deal with it.
7. I've been taking action to try to make the situation better.
8. I've been refusing to believe that it has happened.
9. I've been saying things to let my unpleasant feelings escape. *
10. I’ve been getting help and advice from other people.
11. I've been using alcohol or other drugs to help me get through it.
12. I've been trying to see it in a different light, to make it seem more positive.
13. I’ve been criticizing myself.

14. I've been trying to come up with a strategy about what to do.
15. I've been getting comfort and understanding from someone.
16. I've been giving up the attempt to cope.
17. I've been looking for something good in what is happening.

18. I've been making jokes about it.
19. I've been doing something to think about it less, such as going to movies, watching TV, reading, daydreaming, sleeping, or shopping.
20. I've been accepting the reality of the fact that it has happened.
21. I've been expressing my negative feelings.
22. I've been trying to find comfort in my religion or spiritual beliefs.
23. I’ve been trying to get advice or help from other people about what to do.
24. I've been learning to live with it.
25. I've been thinking hard about what steps to take.
26. I’ve been blaming myself for things that happened.
27. I've been praying or meditating.
28. I've been making fun of the situation.

You can use the 14 types of coping the Carver identifies but Carver recommends users to carry out their own factor analysis to decide the best grouping of items. I did this with approximately 300 nursing students and identified that the 28 items can be grouped into four types of coping. If you are opting for running regressions then it makes sense to use fewer factors to make the regression more manageable. The four types of coping I identified were:

Approach coping	1, 2, 7, 12, 14, 17, 20, 24, 25
Avoidance coping	3, 6, 8, 13, 16, 18, 19, 26, 28
Altering consciousness	4, 11, 22, 27
Seeking support	5, 10, 15, 21, 23

* Item 9 did not load on to any of the four types of coping and so was excluded.

Alternatively you can use Carver's 14 types of coping classifications:

Scales are computed as follows (with no reversals of coding):

Self-distraction, items 1 and 19
Active coping, items 2 and 7
Denial, items 3 and 8
Substance use, items 4 and 11
Use of emotional support, items 5 and 15
Use of instrumental support, items 10 and 23
Behavioral disengagement, items 6 and 16
Venting, items 9 and 21
Positive reframing, items 12 and 17
Planning, items 14 and 25
Humor, items 18 and 28
Acceptance, items 20 and 24
Religion, items 22 and 27
Self-blame, items 13 and 26

Citation: Carver, C. S. (1997). You want to measure coping but your protocol's too long: Consider the Brief COPE. *International Journal of Behavioral Medicine*, 4, 92-100.
