# Supplementary material for: Sense of coherence and strategies for coping with stress among nurses
Source: BMC Nurs. 2021 Jun 23;20:107. doi: 10.1186/s12912-021-00631-1 (PMC8220127; doi:10.1186/s12912-021-00631-1)
Supplement: Supplementary file 3 — Additional file 3. [file 12912_2021_631_MOESM3_ESM.docx]

**Dear Madam / Sir!**

I am asking you to fill in the questionnaires that you have in front of you. The research is anonymous and voluntary. The aim of the research is the specific relationship between the sense of coherence and strategies for coping with stress in a group of professionally active nurses. Thank you for your time and cooperation.

***(Enter the correct answer in the place marked or mark the appropriate square with a cross)***

**SOCIODEMOGRAPHIC DATA**

**1. Gender**

woman

man

**2. Age** …………

**3. Marital status**

free status

married

divorced

separation

widow

concubinage

**4. Place of residence**

village

city ​​up to 50,000 residents

city ​​51-100 thousand residents

city ​​pow. 100 thousand residents

**5. Education**

medical highschool

vocational study

Bachelor of Nursing

Master of Nursing

higher education other (what kind?) ………………………………… ..

**6. Work experience in the profession** …………

**7. Work system**

single shift

two-shift

three-shift

**8. Occupied position at work**

Chief nurse

Ward nurse

Coordinating Nurse

Partial Nurse

other (what?) …………….

**9. How do you assess your financial situation?**

very good

good

neither good nor bad

bad

definitely bad

**10. How do you evaluate your health condition?**

very good

good

neither good nor bad

bad

definitely bad
